# Schlafen2 mutation in mice causes an osteopetrotic phenotype due to a decrease in the number of osteoclast progenitors

**DOI:** 10.1038/s41598-018-31428-z

**Published:** 2018-08-29

**Authors:** Ibrahim Omar, Gali Guterman-Ram, Dolev Rahat, Yuval Tabach, Michael Berger, Noam Levaot

**Affiliations:** 1 The Lautenberg Center for Immunology and Cancer Research, The Biomedical Research Institute Israel Canada of the Faculty of Medicine, The Hebrew University Hadassah Medical School Jerusalem, Jerusalem, Israel; 2 Department of Physiology and Cell Biology, Faculty of Health Sciences, Ben-Gurion University of the Negev, Beer-Sheva, Israel; 3 Department of Developmental Biology and Cancer Research, The Biomedical Research Institute Israel Canada of the Faculty of Medicine, The Hebrew University Hadassah Medical School Jerusalem, Jerusalem, Israel

## Abstract

Osteoclasts are the bone resorbing cells that derive from myeloid progenitor cells. Although there have been recent advancements in the ability to identify osteoclast progenitors, very little is known about the molecular mechanisms governing their homeostasis. Here, by analyzing the normalized phylogenetic profiles of the Schlafen (Slfn) gene family, we found that it co-evolved with osteoclast-related genes. Following these findings, we used a Slfn2 loss-of-function mutant mouse*, elektra*, to study the direct role of Slfn2 in osteoclast development and function. *Slfn2*^*eka/eka*^ mice exhibited a profound increase in their cancellous bone mass and a significant reduction in osteoclast numbers. In addition, monocyte cultures from the bone marrow of *Slfn2*^*eka/eka*^ mice showed a reduction in osteoclast number and total resorption area. Finally, we show that the bone marrow of *Slfn2*^*eka/eka*^ mice have significantly less CD11b^–^Ly6C^hi^ osteoclast precursors. Overall, our data suggest that Slfn2 is required for normal osteoclast differentiation and that loss of its function in mice results in an osteopetrotic phenotype.

## Introduction

Physiological skeletal homeostasis is a well-coordinated process, regulated by the reciprocal actions of bone-forming osteoblasts and bone-resorbing osteoclasts^1,2^. Perturbation of the balance between bone formation and resorption in bone disorders is often mediated by abnormal osteoclast activities^3^. Decreased bone resorption by osteoclasts leads to the formation of sclerotic bone, as seen in osteopetrosis, whereas excessive resorption drives the pathogenesis of osteoporosis, osteoarthritis, periodontal diseases, bone tumor metastasis, as well as multiple bone-related congenital syndromes^3^. Thus, understanding the mechanisms controlling osteoclast number and activity is crucial to the diagnosis and treatment of many clinical conditions.

In hematopoiesis, differentiation of the myeloid-derived osteoclasts requires certain factors, such as macrophage colony-stimulating factor (CSF-1) and the receptor for activation of nuclear factor kappa B Ligand (RANKL)^4–6^, which are produced by marrow stromal cells, osteoblasts, osteocytes, and lymphocytes^7–10^.

Osteoclast-progenitor identification is an emerging topic of a great interest. Previously, several studies have shown that the common monocyte dendritic cell precursor (MDP), which expresses surface CD11b^−^CD115^+^CD117^int^, can differentiate into functioning osteoclasts^11–14^. A more recent study showed that the primary osteoclast precursors (OCP)-containing population in bone marrow is a distinct subset of MDP characterized by CX3CR1^+^ CD11b^−/lo^ Ly6C^hi^ and distinguished from other bone marrow precursors by their pattern of CD11b and Ly6C expression^15^. However, although a great deal is known about how osteoclasts differentiate from precursors and resorb bone, the mechanisms regulating the osteoclast progenitor pool are still elusive.

The Schlafen genes (*Slfn*) were first described in mice as a family transcribed during thymocyte maturation^16,17^. Genomic and phylogenetic studies demonstrated that this family of genes is widely distributed in mammals, where they can be divided into four major clades that experienced lineage-specific expansions or contractions in various orders. In addition, members of the Slfn family been identified in Chondrichthyes and Amphibia, indicating an ancient origin of these genes^18^.

Slfn genes are expressed in tissues of the immune system, and their expression levels vary during T cell and macrophage development as well as in response to infections^16,17^. Evidence indicates a role for Slfn members in the immune response through their function as inhibitors of cell growth or protein translation^16,17^.

The gene encoding Schlafen2 (Slfn2) is highly expressed in all of the cells from the myeloid lineage. Moreover, inflammatory monocyte progenitors in mice harboring the loss-of-function *elektra* allele in Slfn2, undergo apoptosis in response to differentiation signals leading to a severe monocytic-related immunodeficiency^19^. Lee *et al*. observed that Slfn2 expression is induced by RANKL during osteoclastogenesis and that siRNA-mediated downregulation of Slfn2 inhibits this process^20^. These observations suggest that Slfn2’s effects on osteoclast differentiation could be mediated at early stages of monocyte commitment to the osteoclast fate. However, a role for Slfn2 in the regulation of osteoclast precursors and the consequent effects on bone homeostasis were not explored.

Phylogenetic profiling is a comparative genomics method used to identify genes that are functionally related. For some genes, orthologs are found in multiple organisms while others appear in only a handful of species. This pattern of evolution, termed gene phylogenetic profiling, relies on the assumption that proteins that were lost or retained correlatively across millions of years and hundreds of species are probably functionally related. Proteins in a pathway will be conserved in species where their function has an impact on the organism’s fitness. Conversely, in species where the importance of the functionalities performed by the proteins is diminished, the evolutionary conservation is likely to be relaxed. Recently, we successfully developed a phylogenetic profiling approach to study multiple pathways, such as; p53-^21–24^, melanoma-^25,26^ and RNA-associated pathways^27–30^.

In the current study we show that the Slfn genes family co-evolved with osteoclast-related genes. In addition, mice harboring a loss-of-function mutation in Slfn2, *Slfn2*^*eka/eka*^, had a profoundly increased trabecular bone volume fraction as a result of an increase in trabecular numbers and thickness. The bone surface of *Slfn2*^*eka/eka*^ mice had a significant reduction in osteoclast numbers. Furthermore, fewer osteoclasts were generated in bone marrow cultures from *Slfn2*^*eka/eka*^ mice compared to wild type mice. The lower amount of osteoclasts from *Slfn2*^*eka/eka*^ mice translates to a reduction in the total area covered by resorption pits. Finally, we show that bone marrow from *Slfn2*^*eka/eka*^ mice had significantly lower numbers of CD11b^−^Ly6C^hi^ osteoclast precursors. Overall, our data reveal a role for Slfn2 in maintaining the osteoclast progenitor pool, which is essential for proper osteoclast function *in vivo*.

## Results

### Schlafen2 is evolutionary associated with the osteoclast-related genes

**Figure 1:**
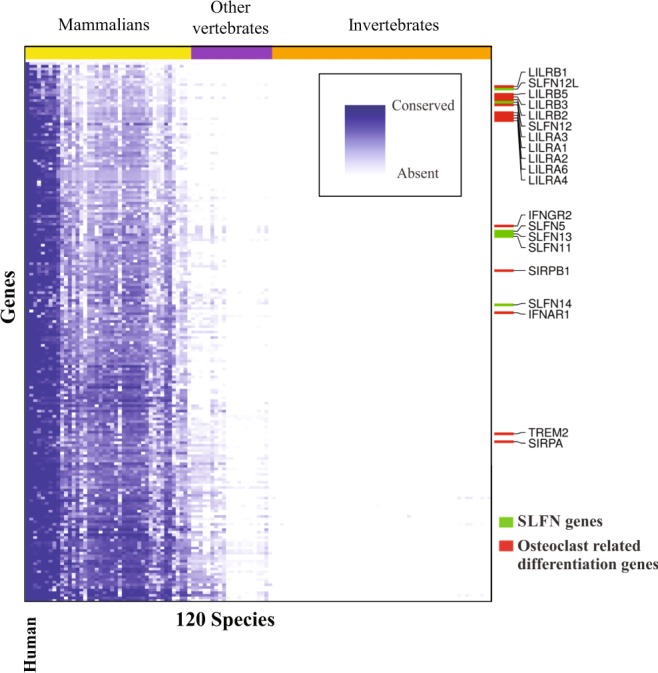
The Schlafen gene family co-evolved with osteoclast differentiation genes. Heatmap presenting genes that are co-evolved with human Slfn genes in different species. Each row in the heatmap represents one of 205 genes found to be co-evolved with at least three Schlafen genes and each column represents a species. The colors in the heatmap indicate the degree of conservation of the gene in the genome of the respective species ranging from white (completely absent) to dark blue (fully conserved). Species are ordered in phylogenetic order and the clade to which each species belongs is indicated in the top color bar.

To identify genes that are functionally related to the SLFN family genes, we generated normalized phylogenetic profiles of 20598 human proteins across 120 animal species as described in our previous studies^25,28^. For this purpose, the phylogenetic profile for each gene, representing its degree of conservation across the species, was generated. For each of the six human SLFN genes (*SLNF5, SLFN11, SLFN12, SLFN12L, SLFN13* and *SLFN14*), we identified the 200 genes whose phylogenetic profiles showed the strongest Pearson correlation. We then focused on genes that co-evolved with three or more of the SLFN genes, resulting in a list of 205 genes (Fig. 1). Notably, SLFN genes are found to be highly correlated with each other (p-value < 10^−12^, hyper-geometric test). Using GeneAnaltyics^31^ to perform enrichment analysis on the list of 205 co-evolved genes, we found the list to be enriched with immune system-related genes (FDR < 10^−12^) and osteoclast differentiation-related genes (FDR < 10^−9^) including *LILRA1, LILRA2, LILRA3, LILRA4, LILRA6, LILRB1, LILRB2, LILRB3, LILRB5, IFNAR1, IFNGR2, SIRPA, SIRPB1* and *TREM2*.

These results suggest that the Slfn gene family may play a role in osteoclast differentiation/function and regulation of bone homeostasis.

### Increased bone mass in *Slfn2*^*eka/eka*^ mice

**Figure 2:**
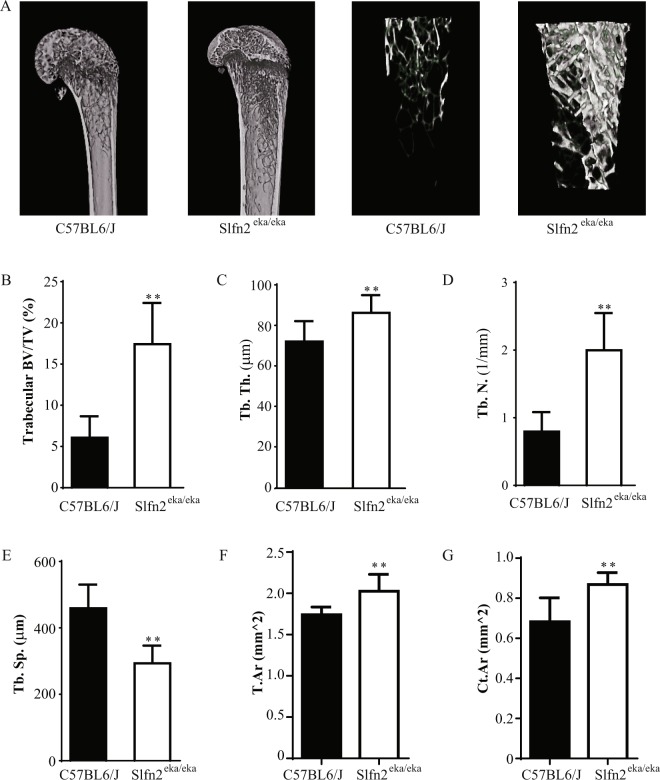
*Slfn2*^*eka/eka*^ mice exhibit increased trabecular and cortical bone phenotype. μCT analysis of trabecular and cortical bone from wild type and *Slfn2*^*eka/eka*^ mice. (**A**) Representative images of µCT scanning. (**B**) Trabecular bone volume (BV.) to total volume (TV.) ratios. (**C**) Trabecular thickness. (**D**) Trabecular numbers. (**E**) Trabecular separation (Tb.Sp.). (**F**) Total tissue area (Tt.Ar). (**G**) Cortical Area (Ct.Ar). *Slfn2*^*eka/eka*^ mice are gender and age matched to wild type mice. n = 10, 10 (wild type, *Slfn2*^*eka/eka*^). P value < 0.01 according to a two-tailed T-test. Results are represented by Mean and SD.

To study the direct role of the Slfn gene family in osteoclast development and function, we utilized our Slfn2 loss-of-function mouse model, *Slfn2*^*eka/eka*^. Initially, we analyzed the bone structure characteristics of these mice. Three-dimensional reconstruction of the femora from 12 week old *Slfn2*^*eka/eka*^ mice using micro-computed tomography (µCT) revealed a profound increase in trabecular bone volume compared to wild type mice (Fig. 2A,B). Trabecular bone volume fraction was 2.91-fold higher in *Slfn2*^*eka/eka*^ mice compared to gender- and age-matched wild type controls as a consequence of higher trabecular thickness and increased numbers of trabeculae (Fig. 2C,D). Trabecular separation was lower in the *Slfn2*^*eka/eka*^ mice compared to controls (Fig. 2E). Analysis of cortical bone showed significantly greater total area and cortical area in *Slfn2*^*eka/eka*^ than in wild type controls (Fig. 2F,G). Overall, these results demonstrate increased bone mass of *Slfn2*^*eka/eka*^ mice.

### Lower osteoclast distribution on *Slfn2*^*eka/eka*^ bone surface

**Figure 3:**
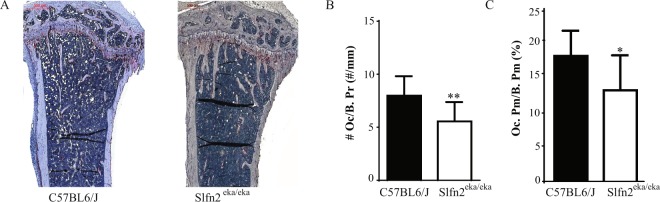
*Slfn2*^*eka/eka*^ mice exhibit a decreased number of osteoclasts. (**A**) Representative images of TRAP (tartrate resistant acid phosphatase) staining in histological slides. (**B**) Measurements of TRAP-stained tibiae; Osteoclast perimeter to bone perimeter. (**C**) Measurements of TRAP-stained tibiae; Osteoclast perimeter to bone surface. Mice were gender and age matched. n = 6. P value < 0.05 according to a two-tailed T-test. Results are represented by Mean and SD.

To elucidate whether the increased bone mass observed in *Slfn2*^*eka/eka*^ mice is due to reduced bone resorption, we analyzed osteoclast distribution in these mice. Histological sections of tibiae from *Slfn2*^*eka/eka*^ mice had greater bone surface compared to wild type controls (Fig. 3A), consistent with the results from the µCT analysis. In addition, histomorphometric analysis revealed decreased osteoclast numbers per bone perimeter (#Oc/B.Pr) and a significant decrease in osteoclast surface relative to total bone surface (Oc.Pr/B.Pm) in *Slfn2*^*eka/eka*^ mice (Fig. 3B,C). These results indicate a developmental defect and/or reduced life span of osteoclast in *Slfn2*^*eka/eka*^ mice.

### Lower osteoclast numbers produced from *Slfn2*^*eka/eka*^ bone marrow *in vitro*

**Figure 4:**
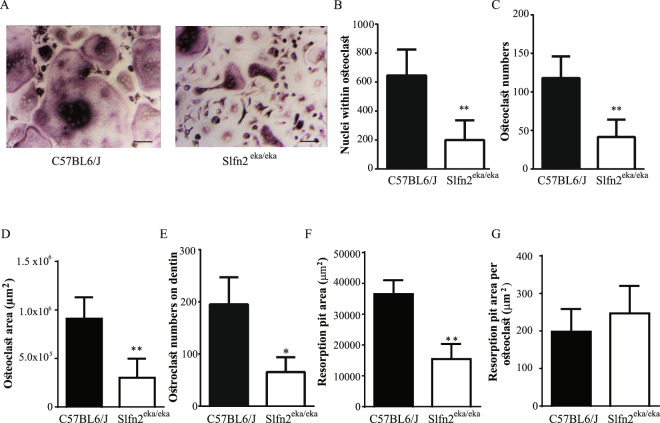
Impaired osteoclast *in vitro* differentiation of *Slfn2*^*eka/eka*^ bone marrow cells. (**A**) Representative images of TRAP-stained osteoclasts in cultures of wild type and *Slfn2*^*eka/eka*^ BMMs (Scale bar = 100 µm). (**B**–**G)** TRAP analysis of: nuclei number within osteoclasts (**B)**, total osteoclast numbers (**C)**, osteoclast surface area (**D)**, total osteoclast numbers on dentin **(E)**, resorption pit area **(F)**, and area of resorption pits per osteoclast (**G)**. Values represent the mean ± SD (**B**–**D** n = 4, 4 and E-G n = 5, 5) with triplicate samples for each mice experiment. *Slfn2*^*eka/eka*^ mice were gender and age matched to wild type mice. P value < 0.05 according to a two-tailed T-test. Results are represented by Mean and SD.

We next examined the capacity of bone marrow-derived monocytes (BMMs) from wild type or *Slfn2*^*eka/eka*^ mice to undergo osteoclastogenesis *in vitro*. BMMs from *Slfn2*^*eka/eka*^ mice cultured on plastic produced almost 70% less osteoclasts, which had a threefold decrease in their surface area and nuclear content (Fig. 4A–D) in comparison to BMMs from wild type mice. Consistent with BMMs cultured on plastic, there was a 66% decrease in osteoclast numbers in cultures of *Slfn2*^*eka/eka*^ BMMs cultured on Dentin discs (Fig. 4E). Measurement of total resorption pit areas showed a 50% reduction in cultures of osteoclasts derived from *Slfn2*^*eka/eka*^ BMMS compare to wild type controls (Fig. 4F). However, when resorption pits were normalized to osteoclast number there was no difference in resorption capacity between *Slfn2*^*eka/eka*^ and wild type osteoclasts (Fig. 4G) indicating the increased total resorption area in these cultures can be attributed to the overall increase in osteoclast numbers.

### Decreased osteoclast progenitor numbers in *Slfn2*^*eka/eka*^ mice

**Figure 5:**
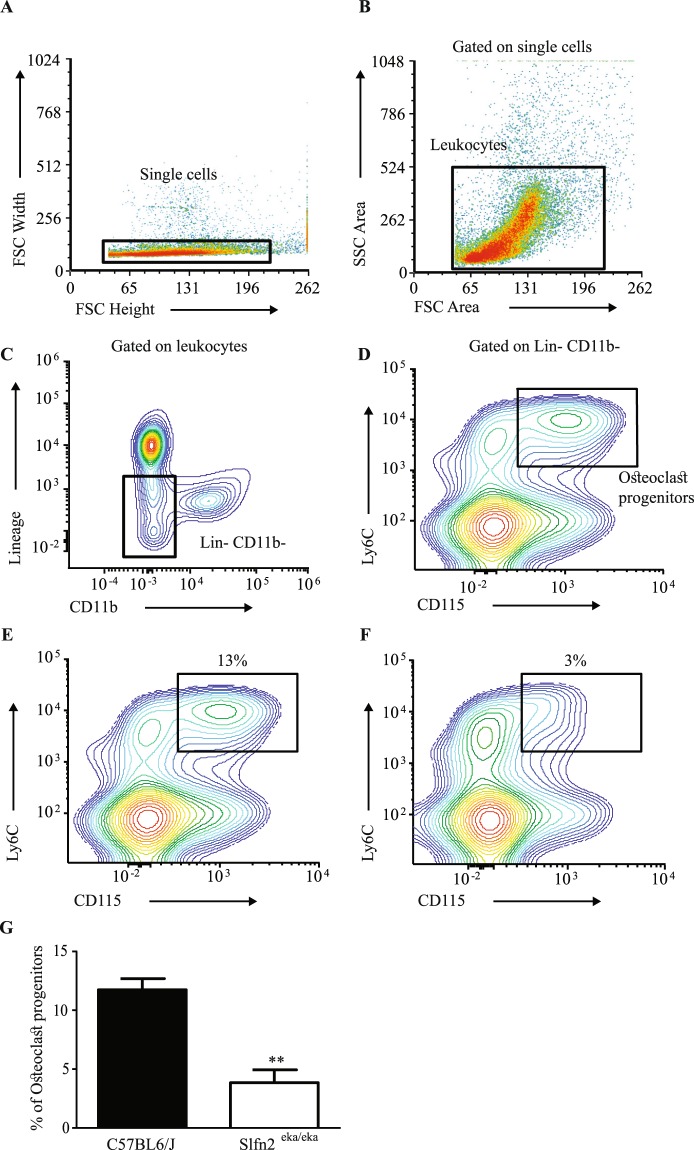
Reduced osteoclast progenitor numbers in *Slfn2*^*eka/eka*^ mice. Bone marrow cells were stained with Biotin anti-mouse lineage antibody cocktail (anti CD3ε, B220, Ly6G and TER119), Pacific Blue conjugated anti-CD11b, Phycoerythrin (PE) conjugated anti-CD115 and Alexa Fluor 700 conjugated anti-Ly6C antibodies. Following the primary stain, cells were stained with Brilliant-violet 510 conjugated streptavidin, and then were subjected to flow cytometry analysis. Primary osteoclast progenitor-containing population. (**A–D**) Gating strategy used to evaluate osteoclast progenitors. (**E,F**) Representative dot plots of osteoclast progenitors population in wild type (**E**) and *Slfn2*^*eka/eka*^ (**F**) bone marrow cells. (**G**) Bar plot summarizing the experiment presented in B (4 mice from each genotype). **P value = 0.0016 (two-tailed Student’s t-test). Error bars are s.e.m.

To further elucidate the cause for reduced osteoclast numbers in *Slfn2*^*eka/eka*^ mice, we analyzed the numbers of osteoclast progenitors. Recently, it has been shown that in mouse bone marrow, lineage negative (CD3ε^−^, B220^−^, Ly6G^−^ and TER119^−^), CD11b^−^, CD115^+^, Ly6C^hi^ cells are the primary osteoclast progenitors (OCP)-containing population^15^. Flow cytometry analysis of BM (Fig. 5A–D) from wild type and *Slfn2*^*eka/eka*^ mice revealed a significant reduction in the percentages of OCP population in *Slfn2*^*eka/eka*^ mice (Fig. 5E–G). These results demonstrate that the OCP population in *Slfn2*^*eka/eka*^ mice is impaired; suggesting that the reduction in osteoclast numbers on *Slfn2*^*eka/eka*^ bone surface is a result of a decrease in the progenitor pool rather than decreased osteoclast differentiation capacity.

## Discussion

We have identified Slfn2 as an important regulator of bone homeostasis. Loss of function of Slfn2 results in a profoundly increased trabecular bone volume fraction, which correlated with fewer osteoclasts on the bone surfaces of *Slfn2*^*eka/eka*^ mice. Several lines of evidence in our study suggest that the lower number of osteoclast on the bone surfaces reflects a decrease in the osteoclast progenitor pool rather than impaired osteoclast differentiation or function. First, there were lower numbers of lineage negative, CD11b^−^, CD115^+^, Ly6C^hi^ osteoclast progenitor cells in the bone marrow of *Slfn2*^*eka/eka*^. Second, fewer osteoclasts were produced *in vitro* from the bone marrow of *Slfn2*^*eka/eka*^ compared to wild type mice. Finally, although the total resorption area was smaller in *Slfn2*^*eka/eka*^ osteoclasts cultures on dentin compared to wild type osteoclast cultures, the resorption area per osteoclast was the same. The profound reduction in osteoclasts strongly suggests that decreased osteoclast function accounts, at least in part, for the high-density bone phenotype observed in *Slfn2*^*eka/eka*^ mice.

We observed a significant increase in cortical area of *Slfn2*^*eka/eka*^ mice. Increased cortical area could reflect disorganized bone modeling during development which is a typical finding in osteopetrotic patients^32^ or osteoclast independent functions of Slfn2 that affect periosteal bone formation. Since loss-of-function of Slfn2 affects other cells than osteoclast progenitors, at this point we cannot rule out indirect mechanisms mediated by the elektra mutation such as effects mediated through other hematopoietic lineage cells or osteoblasts/bone lining cells/osteocytes. Therefore, further investigation is required to determine the overall role of Slfn2 in bone homeostasis.

Disruption of endoplasmic reticulum (ER) homeostasis by pathogenic, physiological or chemical insults leads to the accumulation of unfolded or misfolded proteins in the lumen of the ER, a condition termed ER stress. ER stress stimulates an unfolded protein response (UPR) aimed at restoring ER function. We recently showed that Slfn2 supports the survival of T cells and monocytes by preventing chronic ER stress. Interestingly, it has been shown that ER stress is involved in the differentiation of osteoclast precursor cells and that UPR is induced during osteoclastogenesis. For example, the IRE1α/XBP-1 pathway is transiently activated during osteoclast differentiation and the abrogation of this pathway suppresses osteoclast formation *in vivo* and *in vitro*^25,28^. Despite the critical effect of the ER stress signaling pathway in osteoclast differentiation, chronic unresolved ER stress promotes apoptosis. Therefore, it is possible that the chronic ER stress caused by Slfn2 loss-of-function is the prime mechanism governing the decreased numbers of osteoclast progenitors. In addition, we have recently reported that T cells and monocytes from *Slfn2*^*eka/eka*^ mice have disrupted cholesterol and lipid homeostasis. Since cholesterol is one of the major constituents of biological membranes, several studies showed that it plays an important role in osteoclast formation, fusion and survival^31^. Therefore, cholesterol intracellular levels should be strictly regulated in order to maintain proper osteoclastogenesis. The rate-limiting enzyme of cholesterol synthesis HMG-CoA is upregulated in cells from *Slfn2*^*eka/eka*^ mice leading to elevated *de novo* synthesis of cholesterol in these cells. These data raise the possibility that the defect in osteoclast progenitor homeostasis in *Slfn2*^*eka/eka*^ is due to cholesterol accumulation.

## Material and Methods

### Normalized phylogenetic profiling

A matrix of blastp scores for 20598 human genes against the genomes of 120 animal species was constructed. To avoid false positives due to apparent correlation between genes that are non-conserved along long stretches of species, blastp scores <50 were floored to 50. To avoid biases originating from variability in gene length, the blast score of each gene was normalized to the blast score of the human gene against itself. Further, to avoid biases due to phylogenetic distance, we scaled the conservation score for each species to their overall distribution by transforming the values in the column (corresponding to a species) into z-scores.

The degree of co-evolution between two protein coding genes was then evaluated using the Pearson correlation coefficient between their respective rows in the NPP matrix. When several isoforms of a protein were represented in the matrix, the one achieving the highest correlation coefficient was considered. For each of the human SLFN genes (SLFN5, SLFN11, SLFN12, SLFN12L, SLFN13, SLFN14), we considered the genes with the top 200 correlation coefficients. We then selected genes co-evolved with at least three SLFN genes (n = 205) for further analysis.

Functional enrichment analysis for GO term membership, pathway membership and more was done by submitting the list of 205 coevolved genes to the Gene Analytics web server.

The length normalized phylogenetic profiles of the five SLFN genes and 200 genes that co-evolved with them were ordered using the sorter tool in the sorting points into neighborhoods (SPIN) software^33^, using neighborhood sorting. The ordered profiles were plotted using the gplots package in R, with clustering on both row and columns suppressed.

### Animals

*Slfn2*^*eka/eka*^ mice were previously generated as described in Berger *et al*.^19^. C57BL/6J (wild type) mice were from The Jackson Laboratory. Mice were maintained and bred under specific pathogen free conditions in the Hebrew University’s animal facilities according to the Institutional Animal Care and Use Committee’s regulations. All mouse studies were performed under protocols MD-16-14863 and approved by the Hebrew University Institutional Animal Care and Use Committee. All mice were maintained on the C57BL/6 background and only male 12 week old mice were used in the experiments.

### Microcomputed tomography (μCT)

μCT (SkyScan) was performed on femurs from 12 week old wild type or *Slfn2*^*eka/eka*^ male mice as previously described^34^. Analysis of trabecular bone was performed over 2 mm in length, 0.4 mm below the distal growth plate. Analysis of cortical bone was performed on a region of 1 mm in length in the midshaft. Each image was reconstructed from 200, 11.7 μm slices, 0.4 mm below the growth plate, and trabecular morphometric parameters were determined according to standard protocols. A fixed density threshold was determined using a pair of calibration phantoms (SkyScan).

### Histology

Tibias were fixed in 4% PFA for 48 h. Bones were then decalcified using EDTA for 14 days. Samples were embedded in paraffin for tartrate resistant acid phosphatase (TRAP). Bone analysis was performed by quantifying parameters including osteoclast perimeter/bone perimeter, osteoclast number/bone perimeter and osteoclast perimeter/bone surface. For all analyses, an area 1 mm in height, 0.4 mm below the growth plate and excluding cortical bone was analyzed.

### Isolation and differentiation of bone marrow monocytes

Bone marrow monocytes (BMMs) were harvested from the bone marrow of C57BL6 mice as described previously^35^. Briefly, bone marrow cells were treated with ACK red blood cell lysis buffer (0.15 M NH_4_Cl, 10 mM KHCO_3_, 0.1 mM Na_2_ EDTA in distilled H_2_O). Cells were plated on bacterial culture dishes in α-MEM and 10% FBS for three days in the presence of 20 ng ml^−1^ of CSF-1 and then seeded for various experiments. To induce differentiation, 20 ng ml^−1^ of RANKL (Peprotech) was added to the medium.

### Osteoclast differentiation analysis

At the end point of differentiation, cells were fixed using 4% paraformaldehyde (PFA), and TRAP staining (Sigma-Aldrich) was performed according to the manufacturer’s protocol. Osteoclasts with at least three nuclei were defined as TRAP positive cells. Osteoclast parameters were obtained via the analysis of 20 images from random areas in each well using an Olympus ×83 microscope with an automated stage. Cells in each image were counted in a double-blind manner, and the number of nuclei in the osteoclasts and the total osteoclast surface area were determined using ImageJ software. Each experiment included cells grown in three wells for each sample. The analysis included a total of 600 frames, in which a total of more than 9500 nuclei within osteoclasts were scored for WT mice and more than 2800 nuclei within osteoclasts were counted for *Slfn2*^*eka/eka*^ mice.

### Microscopy

Images were acquired with an inverted IX81 microscope equipped with 20×/0.75 NA objectives (Olympus) and with a temperature-controlled box using CellSens software (Olympus). A PrimoVert microscope equipped with an Axiocam ERc 5 s camera was used to record representative pictures of the cell cultures (ZEISS, Germany).

### Dentin resorption assays

2 × 10^5^ BMMs were plated on dentine discs with RANKL in 12-well plates and cultured for 14 days, with the medium replaced every two days. Osteoclasts were fixed in 3.7% paraformaldehyde, TRAP-stained (Sigma-Aldrich), and counted. Discs were incubated with 0.25 M ammonium hydroxide and mechanically agitated gently for 1 h. The discs were stained with 1% toluidine blue in 1% sodium borate for 5 min, washed with water, and air dried before photographs were taken.

## Data Availability

The datasets generated during and/or analysed during the current study are available from the corresponding author on reasonable request.
